# E2F1 renders prostate cancer cell resistant to ICAM-1 mediated antitumor immunity by NF-κB modulation

**DOI:** 10.1186/1476-4598-13-84

**Published:** 2014-04-17

**Authors:** Zijia Ren, Wenyao Kang, Lihua Wang, Baoliang Sun, Jiajia Ma, Chaogu Zheng, Jie Sun, Zhigang Tian, Xiaoyi Yang, Weihua Xiao

**Affiliations:** 1 CAS Key Laboratory of Innate Immunity and Chronic Disease, Innovation Center for Cell Biology, School of Life Sciences and Medical Center, University of Science and Technology of China, Hefei, Anhui 230027, China; 2 Hefei National Laboratory for Physical Sciences at Microscale, Hefei, Anhui 230027, China; 3 Affiliated Hospital of Weifang Medical College, Shandong 261053, China; 4 Taishan Medical College, Shandong 271000, China

**Keywords:** E2F1, ICAM-1, NF-κB, Short hairpin RNA, Tumor immunity

## Abstract

**Background:**

E2F1 is the gatekeeper of the cell cycle controlling an analogous balance between proliferation and cell death. E2F1 expression is elevated in advanced prostate cancer. However, it is still unclear that the roles and mechanisms of E2F1 on prostate cancers.

**Methods:**

Targeted knockdown by interferon RNA was applied on two prostate cancer and Hela cell lines to examine the inverse correlation expression of E2F1 and ICAM-1. ICAM-1 promoter reporter and ChIP assays were used for analysis of the molecular basis of transcriptional regulation of E2F1 on ICAM-1. Co-IP assays were employed for testing the protein interaction between E2F1 and NF-κB. Tumor xenograft mice model with E2F1 and ICAM-1-knockdown prostate cancer cells were used to investigate the effects of E2F1 and ICAM-1 on antitumor immunity.

**Results:**

E2F1 knockdown by a specific short hairpin RNA increased gene transcription and protein expression of ICAM-1. By using wild type and a series of mutant ICAM-1 promoter luciferase constructs, the NF-κB binding sites were found to be important for E2F1 regulation of ICAM-1 promoter. Targeted knockdown of E2F1 did not affect expression and phosphorylation of NF-κB and IκBα, but facilitated NF-κB binding to the ICAM-1 promoter, subsequently induced ICAM-1 transcription and production in prostate carcinoma cells. Furthermore, knockdown of E2F1 inhibited tumor growth of prostate cancer in vivo through increasing the susceptibility of tumor cells to ICAM-1-mediated anti-tumor immunity including enhancement of monocyte adhesion, leucocytes infiltration, as well as cytotoxicity against tumor cells.

**Conclusions:**

E2F1 knockdown inhibited prostate tumor growth in vitro and in vivo through sensitizing tumor cells to ICAM-1 mediated anti-immunity by NF-κB modulation, highlighting the potential of E2F1 as a therapeutic target.

## Background

Compelling evidences indicate that tumor cells employ mechanisms that circumvent or thwart the immune response to enhance their own growth [1]. Interactions between the immune system and malignant cells play an important role in tumorigenesis [2]. Like most types of cancer, prostate cancer develops in an immune-competent environment. Intercellular adhesion molecule 1 (ICAM-1) is a cell surface glycoprotein in the immunoglobulin superfamily. ICAM-1 has been implicated in enhancing T-cell ability to kill targets because of better cell-to-cell adhesion [3,4]. ICAM-1 can also function to directly costimulate activated T cells [5]. ICAM-1 may be involved in tumor suppression through an immuno-surveillance mechanism [6-9]. It is interesting to explore how transcriptional factors regulate expression of adhesive molecules required for immune cytotoxicity against tumor cells.

E2F1 is an intriguing transcription factor that accumulates the integrated signal of the G1/S transition regulators, required for cell proliferation [10]. Elevated E2F1 protein expression correlated with increased E2F1 mRNA and increased expression of E2F1-target genes DHFR and PCNA, suggesting that E2F1 expression is elevated in advanced prostate cancer [11]. E2F1 induces tumor cell survival via nuclear factor-kappa B (NF-κB) dependent induction of EGR1 transcription in prostate cancer cells [12]. However, it is unknown whether E2F1 affects ICAM-mediated immunity in prostate cancer.

In this study, we show that the expression of ICAM-1 is highly inversely regulated by E2F1 in prostate cell lines. E2F1 knockdown by a specific short hairpin RNA (shRNA) or small interference RNA (siRNA) increased gene transcription and protein expression of ICAM-1 in human prostate cancer cells. Knockdown of E2F1 enhances ICAM-1-mediated peripheral blood mononuclear cells (PBMC) adhesion and cytotoxicity. Furthermore, silencing E2F1 increases ICAM-1 mediated leucocytes infiltration and inhibits tumor growth in vivo. By using wild type and a series of mutant ICAM-1 promoter luciferase constructs, we found that the NF-κB binding sites are important for E2F1 regulation of ICAM-1 through disrupting the formation of p65/p50 heterodimer. These results reveal a novel E2F1 mediated signal circuit for immunoregulation of ICAM-1 beyond its effect on cell cycle. Furthermore, targeted knockdown of E2F1 enhances the susceptibility of tumor cells to ICAM-1-mediated anti-tumor immunity against prostate cancer.

## Results

### ICAM-1 expression is inversely correlated with E2F1 expression by prostate cancer cells

**Figure 1:**
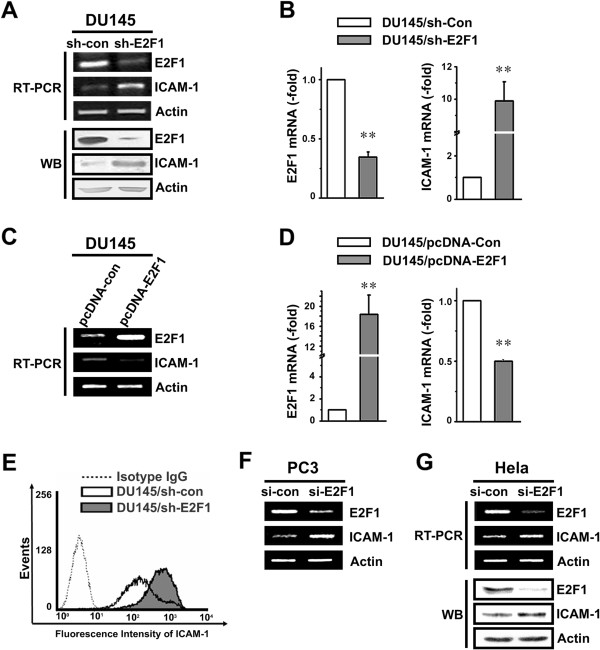
**Expression of ICAM-1 is highly inversely correlated with E2F1 expression. A**. ICAM-1 expression in DU145 cells. Whole cell lysates or total RNA were prepared from DU145 cells stably transfected with either the control vector or shRNA targeting E2F1. The expression of E2F1 and ICAM-1 was monitored by RT-PCR (upper) and Western blot (lower). **B**. ICAM-1 expression in DU145 cells was analyzed by real-time PCR. The stars indicate the significant differences (*P* < 0.05). **C**. E2F1 overexpression plasmids or control were transient transfected to DU145 cells in a 6-well plate for 48 h. The expression of E2F1 and ICAM-1 were measured by RT-PCR. **D**. The expression of E2F1 and ICAM-1 was measured by real time PCR. The stars indicate the significant differences (*P* < 0.05). **E**. Membrane expression of ICAM-1 in DU145/sh-E2F1 and control cells was measured by FACS. **F**. siRNA of E2F1 or scramble control was transient transfected to PC3 cells in a 6-well plate for 48 h. The expression of E2F1 and ICAM-1 in these cells were measured by RT-PCR. **G**. Forty pmol duplex siRNA of E2F1 or scramble control was transient transfected to Hela cells in a 6-well plate for 48 h. The expression of E2F1 and ICAM-1 in these cells were measured by RT-PCR (upper) and Western blotting analysis (lower).

To determine whether expression of ICAM-1 was correlated with the amount of E2F1 in DU145 cells, DU145 cells were stably transfected with pU6 + 27 vectors expressing shRNA of human E2F1. The relative expression of E2F1 was reconfirmed here by reverse transcriptase polymerase chain reaction (RT-PCR) and Western blot (WB) analysis (Figure 1A). The results showed that the level of transcription and expression of ICAM-1 was induced in the DU145 cells with E2F1-knockdown compared with the control cells, which was also confirmed by real-time PCR (Figure 1B). Additionally, overexpression of E2F1 in DU145 cells inhibited the transcription level of ICAM-1 analyzed by RT-PCR (Figure 1C) and real-time PCR (Figure 1D). Expression of membrane ICAM-1 in DU145/sh-E2F1 and control cells was measured by FACS. As shown in Figure 1E, expression of membrane ICAM-1 was induced by E2F1 knockdown in DU145 cells. By interrogating the available microarray data reported by Singh et al. [13], we also found the expression levels of E2F1 and ICAM-1 in prostate samples were inversely correlated, with Spearman’s correlation coefficient of −0.771 (*P* < 0.01) (See Statistical analysis in Methods section).

To further confirm the correlation expression of ICAM-1 and E2F1, another widely used prostate cancer cell line PC3 and Hela cells were transiently transfected with either double strand siRNA-E2F1 or scramble siRNA (si-Con). Indeed, the expression level of ICAM-1 was increased in both PC3 and Hela cells transfected with siRNA-E2F1 when compared with the corresponding control cells transfected with scramble siRNA (Figures 1F and G). These results demonstrated that the expression of ICAM-1 is highly inversely correlated with E2F1 expression in both clinical samples and the prostate cancer cell lines, suggesting that E2F1 may be involved in the transcriptional regulation of ICAM-1.

### E2F1 affects ICAM-1 transcription through the κB sites on ICAM-1 promoter

**Figure 2:**
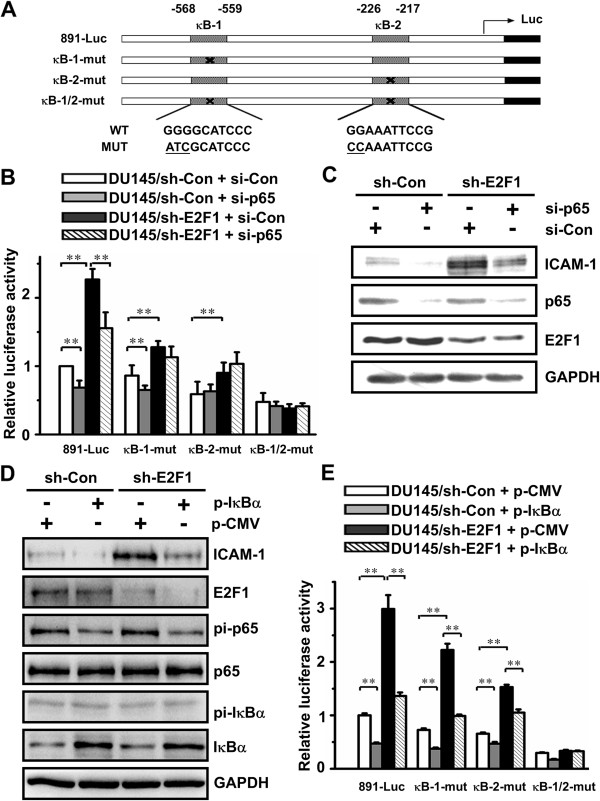
**NF-κB binding sites are required for E2F1 regulation of ICAM-1 promoter. A**. The histograph represent the human ICAM-1 promoter luciferase reporters used in this study. The location of each binding site on the promoter of ICAM-1 is indicated and labeled. The crossed boxes indicate the mutation sites on each construct. The mutated nucleotides corresponding to κB-1-mut and κB-2-mut are underlined. **B** and **C**. DU145/sh-Con and DU145/sh-E2F1 cells were co-transfected with duplex siRNA targeting p65 and either ICAM-1 wild type or mutant promoter reporters. The stars indicate the significant differences (*P* < 0.05) **(B)**. The expression of membrane ICAM-1 was measured by Western blot **(C)**. The data are shown as the mean ± SD of triplicate measurements. **D** and **E**. DU145/sh-Con and DU145/sh-E2F1 cells were transiently transfected with either the empty vector or the mutant IκBα expressing plasmids. The phosphorylation of p65 and IκBα and the expression of ICAM-1 in these indicated cells were monitered by Western blot **(D)**. DU145/sh-Con and DU145/sh-E2F1 cells were co-transfected with IκBα expressing plasmids and either the ICAM-1 wild type or mutant promoter reporters for the luciferase activity assay **(E)**. The results are shown as the mean ± SD of triplicate measurements. The stars indicate the significant differences (*P* < 0.05).

Based on a computational analysis of ICAM-1 promoter, there is no E2F1 binding motif in the promoter of the ICAM-1 gene. Previous studies have implicated that ICAM-1 can be transcriptionally up-regulated by NF-κB through the proximal NF-κB regulatory region located between −226 to −217 (5′-GGAAATTCCG-3′) [14,15]. Moreover, it was revealed that there was another potential NF-κB site at positions −568 to −559 (5′-GGGGCATCCC-3′) [16]. These two NF-κB sites were then designated as κB-1 (−568 to −559) and κB-2 (−226 to −217) (Figure 2A).

To explore how E2F1 could transcriptional regulate expression of ICAM-1, the wild type and a series of mutant ICAM-1 promoter luciferase were constructed and used to assess whether E2F1 affects activation of ICAM-1 promoter mediated by the κB sites. A human ICAM-1 promoter (from −891 to −58 upstream of ATG codon) was cloned from human genomic DNA by PCR and inserted into the pGL3-basic vector (891-Luc). ICAM-1 promoter mutation constructs designated as κB-1-mut and κB-2-mut containing point mutations in each corresponding potential NF-κB binding site were created using site directed mutagenesis. In addition, the ICAM-1 promoter reporter containing both mutation of κB-1 and κB-2 binding site was also constructed and designated as κB-1/2-mut (Figure 2A). DU145/sh-Con and DU145/sh-E2F1 cells were transiently transfected with the ICAM-1 promoter constructs for 48 h and assayed for luciferase activity. As shown in Figure 2B, the activity of the wild type 891-Luc promoter in DU145/sh-E2F1 cells was 2.3-fold higher than in DU145/sh-Con cells, demonstrating that E2F1 knockdown increased ICAM-1 transactivation. Either mutation at κB-1 or κB-2 binding site equally reduced the regulation from 2.3- to 1.5-fold in these reporter genes. The double mutated κB-1/2 reporter gene had a lower level luciferase activity than that of the wild-type construct and was scarcely affected by knockdown of E2F1. These results indicated that E2F1-regulating suppression of ICAM-1 requires both κB-1 and κB-2 sites.

### E2F1 regulates the expression of endogenous ICAM-1 under the conditional loss or inactivation of NF-κB

Having asserted the importance of the NF-κB binding sites for ICAM-1 gene expression, we next determined whether loss or inactivation of NF-κB affected the expression of endogenous ICAM-1. Therefore, we knocked down the expression of NF-κB p65 subunit using double-strand siRNA in DU145 cells. Loss of expression of p65 was confirmed by Western blot (Figure 2C). Knockdown of p65 resulted in a dramatically decreased expression of endogenous ICAM-1 and E2F1 knockdown-induced ICAM-1, indicating a requirement for NF-κB in E2F1 regulating expression of ICAM-1.

NF-κB inhibitor alpha (IκBα) inhibits NF-κB by masking the nuclear localization signals of NF-κB proteins and keeping them sequestered in an inactive state in the cytoplasm [17]. To functionally inactivate NF-κB, a pCMV-IκBαM plasmid which contains two mutations that prevent the phosphorylation step of IκBα was introduced into DU145/sh-Con and DU145/sh-E2F1 cells. The expression and activation of IκBα and NF-κB p65 were confirmed by Western blot (Figure 2D). Overexpression of mutant IκBα suppressed the level of phosphorylation of p65 and the expression of ICAM-1. The increase of ICAM-1 expression induced by E2F1 knockdown was abolished partially by overexpressing IκBα, though neither the level of expression nor phosphorylation of NF-κB and IκBα was affected by knockdown of E2F1.

Next, the effect of E2F1 knockdown on the transactivation of ICAM-1 promoter was examined under the conditional loss or inactivation of NF-κB (Figures 2B and E). When DU145/sh-Con and DU145/sh-E2F1 cells were transfected with duplex siRNA of NF-κB/p65 (Figure 2B), the transactivation of the wild type ICAM-1 promoter induced by E2F1 knockdown was significantly reduced in the case of the decreased p65 level; Similar results were obtained from the above cells overexpressing IκBα through transfection of pCMV-IκBαM plasmid (Figure 2E). The activity of κB-1/2-mut was not induced in any of these cells. Thus, the NF-κB is required for E2F1 regulating transcription of ICAM-1.

### E2F1 affects NF-κB binding to ICAM-1 promoter

**Figure 3:**
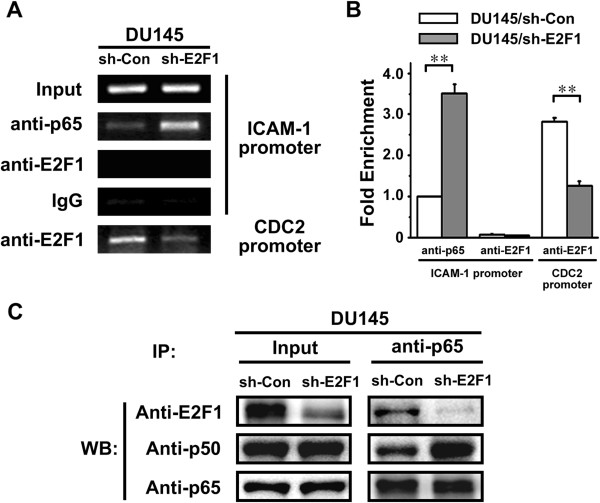
**RNA Silencing of E2F1 increases p65/p50 heterdimer binding to ICAM-1 promoter. A**. ChIP assay was performed with cell lysates from DU145/sh-Con and DU145/sh-E2F1 cells. The chromatin was immunoprecipitated with anti-E2F1 and anti-p65 antibodies or normal IgG which served as a negative control. A pair of primers flanking the κB-1 binding site within the ICAM-1 promoter was used in PCR. PCR for the E2F1 binding site within the CDC2 promoter served as a positive control for detecting E2F1 binding activity. **B**. Real-time PCR was employed to the ChIP assay in (A). Relative occupancy values were calculated by determining the apparent immunoprecipitation efficiency (ratios of the amount of immunoprecipitated DNA to that of the input sample) and normalized to the level of p65 binding with ICAM-1 promoter in DU145/sh-Con cells, which was defined as 1.0. **C**. The relationship among E2F1 and p65/p50 heterodimer in DU145/sh-Con and DU145/sh-E2F1 cells was examined by coimmunoprecipitation analysis. Anti-p65 antibody was used for immunoprecipitation. The amounts of E2F1, p65 and p50 in the immunoprecipitates were detected by Western blot with the indicated specific antibodies.

To access whether E2F1 regulates NF-κB binding to the NF-κB site on ICAM-1 promoter, a chromatin immunoprecipitation (ChIP) assay was performed (Figures 3A and B). The cell lysates from DU145/sh-Con and DU145/sh-E2F1 cells were immunoprecipitated with anti-E2F1 and anti-p65 antibodies or normal IgG. A pair of primers flanking the κB binding sites within the ICAM-1 promoter was used in conventional PCR (Figure 3A) and real-time PCR (Figure 3B). PCR for the E2F1 binding site within the CDC2 promoter served as a positive control for detecting E2F1 binding activity [18]. As expected, E2F1 did not bind directly to the κB sites within the ICAM-1 promoter. Nevertheless, NF-κB bound to its specific binding sites and particularly it was significantly increased when E2F1 was depleted in DU145/sh-E2F1. These data strongly suggested that E2F1 may serve as a sequester of NF-κB p65 to prevent it from binding to the ICAM-1 promoter. Thus, knockdown of E2F1 increases NF-κB binding to ICAM-1 promoter, resulting in up-regulation of ICAM-1 transcription.

### E2F1 interacts with NF-κB p65/p50 heterodimer

To determine how E2F1 prevents NF-κB p65 from binding to the ICAM-1 promoter, we tested the complex formation between E2F1 and NF-κB in a co-immunoprecipitation experiment. Cell lysates from DU145/sh-E2F1 and its parent DU145/sh-Con cells were prepared and immunoprecipated with an anti-NF-κB p65 monoclonal antibody; immunoprecipitates were blotted with anti-NF-κB p65, −NF-κB p50 and anti-E2F1. As shown in Figure 3C, not only did NF-κB p65 form a complex with NF-κB p50 or NF-κB p65, but also it associated with E2F1 in DU145/sh-Con cells, demonstrating that a direct physical protein-protein interaction occurs between E2F-1 and NF-κB. Indeed, the NF-κB p65/p50 complex was remarkably increased in DU145/sh-E2F1. These data revealed that E2F1 acts as a suppressor to interfere with NF-κB p65 association with NF-κB p50; knockdown of E2F1 could enhance formation of NF-κB p65/p50 heterodimer, resulting in facilitating NF-κB p65 binding to the ICAM-1 promoter and up-regulation of ICAM-1 transcription.

### E2F1 knockdown enhances cell adhesion through ICAM-1

**Figure 4:**
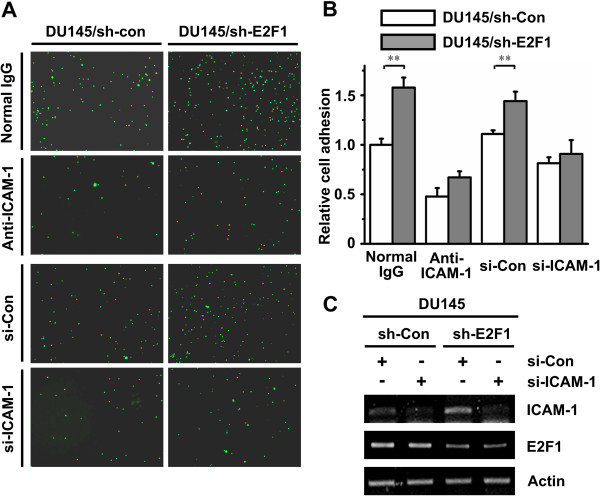
**E2F1 knockdown enhances cell adhesion through ICAM-1. A**. DU145/sh-Con and DU145/sh-E2F1 cells were pre-incubated with anti-ICAM-1 antibody or transient transfected with duplex siRNA of ICAM-1. PBMC labeled with Calcein-AM were added to the indicated cells. After washing three times, attached PBMC were captured using a fluorescence microscope at nine randomly selected fields of each well. **B**. The number of attached PBMC in the **(A)** was presented by the bar graph (white bar, DU145/sh-Con; gray bar, DU145/sh-E2F1). The data are shown as the mean value ± SD of triplicate measurement. The stars indicate the significant differences (*P* < 0.05). **C**. RT-PCR was employed to validate the efficiency of the siRNA-ICAM-1 in DU145/sh-Con and DU145/sh-E2F1 cells that were used in **(A)**.

It was reported that interaction between ICAM-1 and its counter-receptor lymphocyte function-associated antigen (LFA-1) plays a role in immune cell mediated host defense mechanisms [19,20]. To study the effect of E2F1 regulating ICAM-1 on the binding of monocyte to DU145 cells, cell adhesion assay was performed. The number of the adhesive peripheral blood mononuclear cells (PBMC) was increased in DU145/sh-E2F1 cells compared to control cells (Figures 4A and B). The induction of adhesion by knockdown of E2F1 was impaired when the ICAM-1 antibody was pre-incubated with the cells to block ICAM-1 on cell surface or the expression of ICAM-1 was knocked down by its specific siRNA (Figure 4C). These results indicated E2F1 knockdown could enhance PBMC adhesion to DU145 cells through up-regulating ICAM-1.

### E2F1 knockdown increases cytotoxicity of cytokine-induced killer cells against prostate cancer cells, correlating with surface expression of ICAM-1

**Figure 5:**
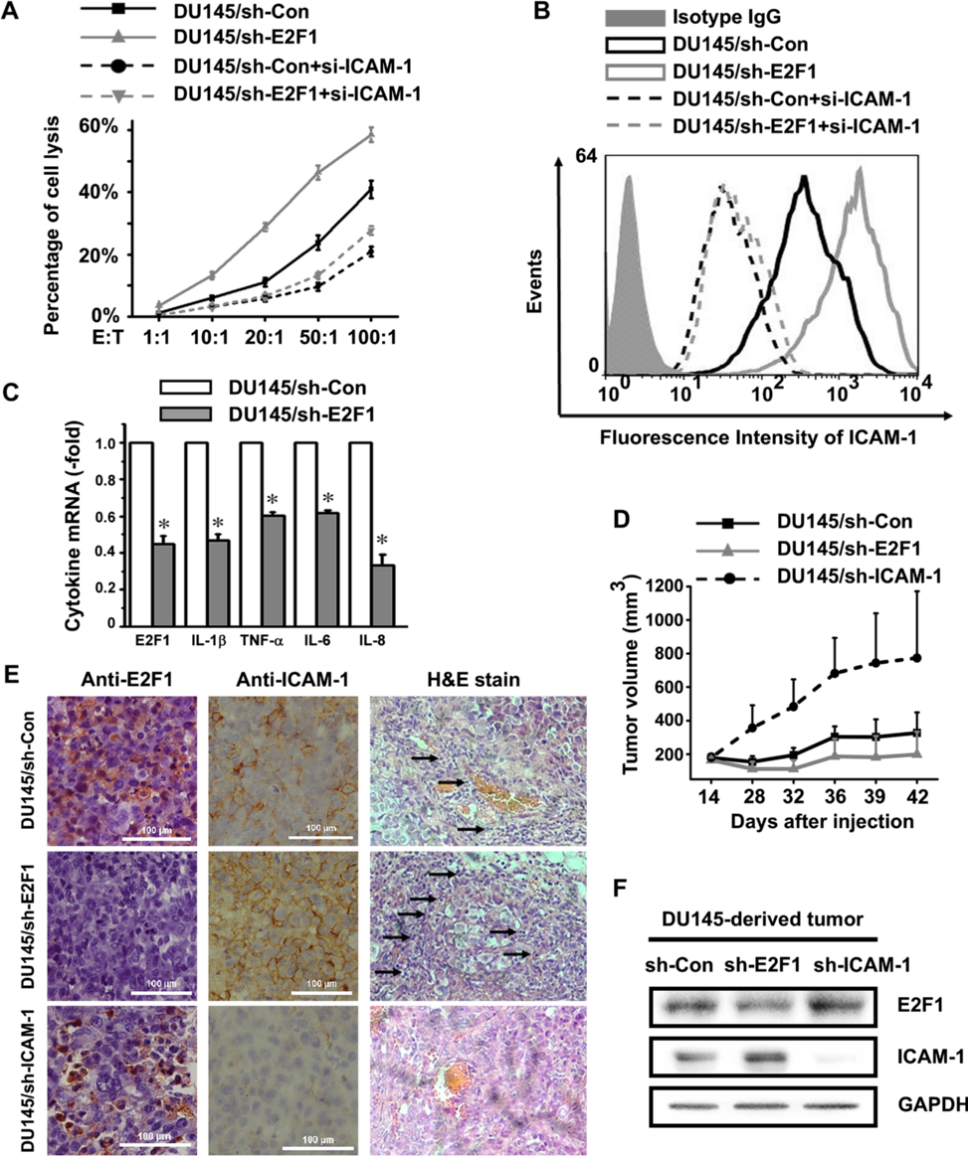
**RNA Silencing of E2F1 Sensitizes Prostate Cancer Cells to ICAM-1 Mediated Anti-tumor Responses. A**. E2F1 knockdown increases cytotoxicity through ICAM-1. DU145/sh-Con and DU145/sh-E2F1 cells were transiently transfected with duplex siRNA of ICAM-1. The DU145 derived cells were used as the target cells and the CIK cells as the effector cells. Both of them at various ratios of effector cells to target cells were mixed and cytotoxicity was measured by ^51^Cr release. The results are shown as the mean ± SD of triplicate measurements. **B**. The expression of membrane ICAM-1 in the indicated cells was measured by FACS with ICAM-1 specific antibody. **C**. The expression of IL-1β, TNF-α, IL-6 and IL-8 in DU145/sh-Con and DU145/sh-E2F1 cells were measured by real-time PCR. The data presents the fold-induction of the levels of each tested cytokine in DU145/sh-E2F1 cells over DU145/sh-Con cell, and represent the mean ± SD of triplicate measurements. **D**. E2F1 knockdown inhibits tumor xenografts growth in vivo, DU145 cells were stably transfected with the control vectors or shRNA expressing plasmids targeting E2F1 or ICAM-1 respectively and injected subcutaneously into nude mice. Tumor volumes were measured and estimated by the formula: length (mm) X width (mm) X height (mm)/2. The results are shown as the mean value ± SD of at least five tumors in each group. **E** and **F**. E2F1 knockdown increases ICAM-1-mediated leucocytes infiltration. The infiltrating leucocytes to DU145-derived tumors in nude mice were stained by H&E and indicated by arrows. The expression of E2F1 and ICAM-1 in these DU145-derived tumors was monitored by immunohistochemistry analysis, H&E staining **(D)** and Western blot **(E)**.

Cytokine-induced killer (CIK) cells are highly efficient cytotoxic effector cells capable of lysing tumor cell targets. The cytotoxic effect of CIK cells against tumor targets is blocked by antibodies directed against LFA-1 and ICAM-1 [21-23]. The CIK cell subpopulations were tested for cytotoxicity against the prostate cancer cells as measured by ^51^Cr release. The DU145 derived cells were used as the target cells and the CIK cells as the effector cells. Both of them at various ratios of effector cells to target cells were mixed together for evaluating the effect of E2F1 in regulating ICAM-1 on the CIK cell cytotoxicity to DU145 cells. Figure 5A shows the cytotoxicity of effector cells against DU145 derived prostate cancer cells. DU145/sh-E2F1 cells showed a higher rate of cell lysis than DU145/sh-Con cells after co-incubation with CIK cells for 4 h. DU145/sh-E2F1 cells were found to be more susceptible to cytolysis by CIK cells than DU145/sh-Con. As expected, the loss of cell cytolysis was observed in DU145/si-ICAM-1 cells. Furthermore, the induction of cytolysis induced by E2F1 knockdown was significantly counteracted by specific siRNA of ICAM-1, shown in DU145/sh-E2F1 + si-ICAM-1 cells. These data demonstrated that E2F1-mediated increase in ICAM-1 levels plays a role in killing tumor cells, which may represent another mechanism of the involvement of E2F1 in tumor growth beyond its effects in cell proliferation.

To get more insight into the potential role of ICAM-1 in E2F1 controlling target tumor cells susceptibility to CIK cytolysis, the expression level of ICAM-1 on the surface of DU145 derived prostate cancer cells was examined by using flow cytometric analysis (Figure 5B). As expected, little or no expression of ICAM-1 was observed on the surface of DU145/si-ICAM-1 cells. DU145/sh-E2F1 cells showed a higher level of expression of ICAM-1 compared to DU145/sh-Con cells, indicating knockdown of E2F1 increased ICAM-1 expression on the surface of DU145 cells. These data suggested that the lytic capacity of CIK cells correlates with the expression level of ICAM-1 in DU145-derived cells modulated by E2F1.

The expression of inflammation cytokines is majorly regulated by NF-κB and has been indicated in promoting tumor growth and survival. Since this study revealed that E2F1 inhibited NF-κB transactivation activity on ICAM-1 expression, it is interesting to examine whether E2F1 affects the cytokine expressions in the same cell model. As shown in Figure 5C, the inflammation cytokines including IL-1β, TNF-α, IL-6 and IL-8 were significantly decreased in DU145/sh-E2F1 cells compared with DU145/sh-Con cells. Although the major sources of inflammation cytokines in tumor microenviroment are coming from the infiltrated immune cells, the results implicated that the reduction of these cytokines in DU145/sh-E2F1 may also contribute to the inhibition of the tumor cell growth. Furthermore, in contrast with the inhibitory action of E2F1 on NF-κB regulated ICAM-1 transcription (Figures 1, 2 and 3), knockdown of E2F1 suppressed the expression of the tested cytokines (Figure 5C), revealing that E2F1 plays different roles on the transcriptional activities of NF-κB in an regulatory sequence specific manner, which is consistent with the previously reports [24].

### E2F1 knockdown increases ICAM-1 expression and suppresses growth of prostate tumor xenografts in a mouse model

The in vivo effect of E2F1 and ICAM-1 expression on DU145-derived tumor growth was evaluated in the xenograft mouse model. DU145 cells were stably transfected with pU6 + 27/shRNA-ICAM-1 plasmids to generate DU145/sh-ICAM-1 cell lines. Then, DU145/sh-ICAM-1, DU145/sh-E2F1 and control cells were injected subcutaneously into nude mice respectively. As shown in Figure 5D, the tumor sizes following inoculation of DU145/sh-E2F1 cells were smaller than control cells. However, the size of tumors derived from DU145/sh-ICAM-1 cells was significantly larger than control. Such difference in xenografts developed from DU145/sh-E2F1 and DU145/sh-ICAM-1 cells demonstrated the opposing effects between E2F1 and ICAM-1 in tumorigenesis of prostate cancer. More importantly, E2F1 knockdown could inhibit growth of prostate tumor xenografts in vivo.

Although E2F1 may affect tumor growth through regulating cell cycle or apoptosis, it is interesting to explore whether there is another mechanism of the involvement of E2F1 in tumor growth. One critical aspect of our study was to examine whether knockdown of E2F1 in DU145 cell-derived tumor tissues is able to increase expression of ICAM-1 and ICAM-1-mediated leucocytes infiltration in vivo. We performed Western blot and immunohistochemistry analysis to evaluate the expression of ICAM-1 and E2F1 in the indicated tumor tissues. The level of E2F1 in DU145/sh-E2F1 tumors was reduced compared to DU145/sh-Con. It was observed that expression of ICAM-1 in DU145/sh-E2F1 tumors was higher than control and DU145/sh-ICAM-1 tumors (Figures 5E and F), indicating that the increase in ICAM-1 expression is correlated to suppression of DU145 xenograft tumor growth by E2F1 knockdown.

### E2F1 knockdown increases ICAM-1-mediated leucocytes infiltration in vivo

Tumor infiltration of immune cells mediated by ICAM-1 was thought to be an attempt by the host organism to combat malignancy [25]. Therefore we observed the effect of E2F1 knockdown on ICAM-1-mediated leucocytes infiltration in vivo. The histological observations by Hematoxylin and eosin (H&E) staining (Figure 5E) showed that there were no or few leukocytes infiltrated into the DU145/sh-ICAM-1 tumors. More leukocytes were infiltrating into the DU145/sh-E2F1 tumors than into the DU145/sh-Con tumors. Expression of ICAM-1 in tumor tissues was also studied by using immunohistochemistry method. A very weak signal was observed in DU145/sh-ICAM-1 tumor tissues. ICAM-1 staining in DU145/sh-E2F1 tumor tissues was remarkably higher than that in DU145/sh-Con, consistent with the data obtained from Western blot assay (Figure 5F). These results suggested that knockdown of E2F1 could increase on-site leucocytes infiltration to tumors in vivo, which correlated with expression of ICAM-1.

## Discussion

Tumors use multiple mechanisms to escape from immune-mediated rejection. ICAM-1 has been considered as not only an adhesion molecule but also a co-stimulatory molecule that provides signal to cytotoxic T lymphocytes (CTL) and natural killer (NK) cells. ICAM-1 has been associated with cellular migration into inflammatory sites and with facilitating interactions between lymphocytes and tumor targets in the pathway of cell-mediated cytotoxicity [25]. Previous murine studies have shown that the introduction of the ICAM-1 gene into tumor cells using retroviral vectors led to enhanced antitumor responses [5]. Furthermore, numerous studies have documented a link between the immune infiltrate and response to therapy [26,27]. Vesalainen et al. found that low numbers of tumor-infiltrating lymphocytes were a sign of high risk of tumor progression and fatal disease in an analysis with 325 cases with long-term follow-up [28]. In contrast, Kärjä et al. found that a strong expression of intratumoral T-cells and B-cells was an independent predictor of shortened PSA recurrence-free survival [29]. Our data shows that E2F1 knockdown increased enhanced leucocytes infiltration into tumor tissues and inhibited tumor growth in vivo. It is interesting to note that the effect of E2F1 knockdown are being evaluated with human cells in vitro and employing xenograft models, i.e., in a host without an intact immune system. Most of the mononuclear cells were murine NK cells due to the lack of T cells in the nude mouse. Human ICAM-1 has a 53% structural homology with the murine variant. It has been reported that murine LFA-1 on NK cells adheres to human ICAM-1 on tumor cells and provides an anti-tumor effect, and ICAM-1 plays an important role in cytolysis by NK cells [5,20]. We also observed cytotoxicity of NK cells to cancer cells was enhanced by increasing ICAM-1 mediated by E2F1 knockdown (Figure 5A). This suggests that the effects of ICAM-1 upregulated by E2F1 knockdown may have important implications for the systemic treatment of prostate cancer.

Tanaka et al. found that E2F1 could bind to p65 in competition with p50, inhibit the formation of functional p65/p50 heterodimer and suppress the transcription of MnSOD in murine fibroblasts [30]. It has been reported that p65/p50 heterodimer binds with the κB site on ICAM-1 promoter, and actives the expression of ICAM-1 [14]. Our studies showed that the interaction of E2F1 and p65/p50 also affects the expression of ICAM-1. E2F1 acts as a suppressor to prevent p65/p50 heterodimer from binding to ICAM-1 promoter. However, instead of inhibiting gene transcription, Lim et al. reported E2F1 could act as a transcriptional activator recruited by NF-κB in response to LPS [24]. Our previous studies also demonstrated that E2F1 stimulated the transcriptional activity of NF-κB on the EGR1 promoter [12]. The significant difference between the regulation of EGR1 and ICAM-1 by E2F1 is the binding of E2F1 with the target gene promoter. E2F1 binds to the promoter of EGR1 but not ICAM-1 (Figure 3). Indeed, a panel of inflammation cytokines including IL-1β, TNF-α, IL-6 and IL-8 are down-regulated when E2F1 is absented (Figure 5C). These suggest that E2F1 can positively or negatively regulates NF-κB activity, depending on the promoter of the target genes.

**Figure 6:**
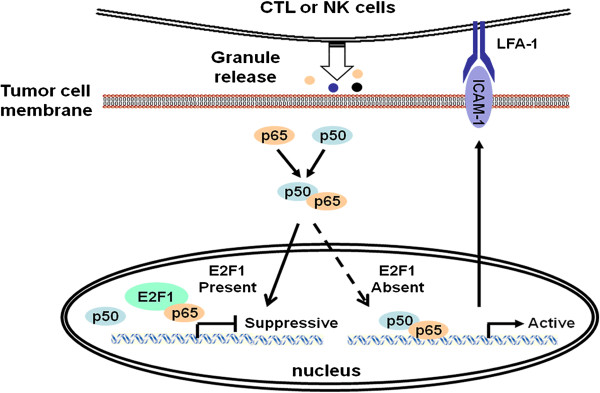
**A schematic model whereby E2F1 regulates the ICAM-1 mediated anti-tumor immune circuit through NF-κB modulation.** NF-κB binding site is required for E2F1 regulation of ICAM-1. E2F1 interacts with NF-κB forming an NF-κB/E2F1 complex. E2F1 acts as a suppressor to prevent the NF-κB p65/p50 complex from binding to ICAM-1 promoter. As a consequence, E2F1 interferes with the adhesion of monocytes onto prostate cancer cells, the sensitivity of tumor cells to the cytotoxic effect of cytokine-induced killer cells and the growth of prostate cancer cells. Targeted knockdown of E2F1 does not affect the expression of phosphorylation of NF-κB p65 and IκBα, but releases NF-κB p65 to facilitate p65/p50 heterodimerization and binding to the ICAM-1 promoter. Subsequently, ICAM-1 transcription and production are induced, resulting in the enhanced immune cells mediated cytotoxicity against prostate carcinoma cells.

Our studies support a model whereby for E2F1 regulation of tumor immune escape mediated by suppressing ICAM-1 (Figure 6). NF-κB binding sites are important regulatory elements for E2F1 regulation of ICAM-1 (Figure 2). E2F1 interacts with NF-κB forming an E2F1/NF-κB complex. Such molecular interplay between E2F1 and NF-κB in the regulation of ICAM-1 is dependent on p65. E2F1 acts as a suppressor to prevent the NF-κB p65/p50 complex from binding to ICAM-1 promoter. When E2F1 is targeted knockdown by shRNA-E2F1, which does not affect expression and phosphorylation of NF-κB/p65 and IκBα, but facilitates NF-κB binding to the ICAM-1 promoter (Figure 3), ICAM-1 transcription and production are subsequently induced, resulting in antitumor immunity against prostate carcinoma cells.

## Conclusions

Taken together, our data provided the evidence that E2F1 influences the ICAM-1 mediated anti-tumor microenvironment immune circuit through NF-κB modulation, which regulates prostate cancer cells escape from immune responses. A functional interaction between E2F1 and NF-κB regulates ICAM-1 transcription and production. Targeting knockdown of E2F1 resulted in the induction of ICAM-1 production and ICAM-mediated anti-tumor immunity to prostate cancer cells, highlighting a new functional signal circuit of E2F1 on immunoregulation of ICAM-1 beyond cell cycle and the potential of E2F1 knockdown act as an effective therapeutic approach against prostate cancer.

## Methods

### Reagents

Cell culture components were purchased from Gibco. The following specific antibodies were used in this study: anti-p65, anti-p50, and anti-E2F1 polyclonal antibodies (Santa Cruz Biotech); anti-ICAM-1, anti-IκBα, anti-GAPDH, anti-phosphorylated p65 (p-Ser276), and anti-phosphorylated IkBα (p-Ser32/36) monoclonal antibodies (Cell Signaling); and PE mouse anti-human ICAM-1 antibody (BD Biosciences). pCMV-IκBαM plasmid was purchased from Clontech. pcDNA-E2F1 plasmid was a gift from Dr. Mian Wu. All other reagents and chemicals used for this study were purchased from Sigma-Aldrich, Inc.

### Short hairpin RNA (shRNA) expression and small interfering RNA (siRNA) duplex

Two types of RNA interference were used to knockdown the expression of E2F1 and ICAM-1, which were shRNA expression plasmids and duplex siRNA. The pU6 + 27/shRNA expression vectors of human E2F1 were constructed as described in previous study, which targeting amino acids 125 to 131 of human E2F1 [12].

### Cell culture and transfection

DU145, PC3 and Hela cells were purchased from American Type Culture Collection (ATCC) and cultured at 37°C with 5% CO_2_ in complete DMEM (Gibco) supplemented with 10% fetal bovine serum (FBS) and 2 mmol/L of L-glutamine. Lipofectamine 2000 (Invitrogen Corporation) was used for transfection. Stably transfected cell lines with pU6 + 27/shRNA expression vectors were obtained after being selectively screened by G418 (800 μg/ml).

### Construction of luciferase reporter plasmids

The ICAM-1 promoter region (bp −897 to −58 upstream of the translation initiation site) was amplified by polymerase chain reaction using human genomic DNA (Clontech) as a template with the primers ICAM-1(−897): 5′-AGG GAG CTC TCG TCA AGA TCC AAG CTA GCT G-3′ and ICAM-1(−58): 5′-GGA AGA TCT GTG ATC CTT TAT AGC GCT AGC C-3′. A series of luciferase reporters were then constructed by site-directed PCR mutagenesis using the 897-luc plasmid as the template and a set of primers containing the corresponding point mutations on the NF-κB binding sites. The primers for generating the point mutation on the NF-κB binding sites are: 5′- GAG GGA TGC *GAT* TCC CCC TAG GTC ACG TCC AC -3′ and 5′ – TAG GGG GA*A TC*G CAT CCC TCA GTG GAG GGA G -3′ for NF-κB binding site-1; 5′- TGG CCG CTT CAG CTC CGG AAT TT*G G*AA GC -3′ and 5′- GCC GCC CGA TTG CTT TAG CTT *CC*A AAT TCC -3′ for NF-κB binding site-2. The point mutations are indicated as *Italics*.

### Flow cytometry analysis

The cells were washed with PBS and incubated with PE conjugated anti-ICAM-1 antibody for 30 min. An aliquot of 10^4^ cells was subsequently analyzed by FACSCalibur (BD Biosciences).

### Reverse transcription-PCR and Real-time PCR

Isolated total RNA was subjected to cDNA synthesis by Superscript III reverse transcriptase (Invitrogen). PCR products were quantitated by agarose gel electrophoresis or real-time PCR using an ABI7500 instrument (Applied Biosystems) and FastStart Sybr Green Master kit (Roche). The following primer sets were used for PCR: humanE2F1-RT-sense: 5′-GTC ACG CTA TGA GAC CTC AC-3′, humanE2F1-RT-antisense: 5′-TCA AGG ACG TTG GTG ATG TC-3′; beta-actin-sense: 5′-GAC CTG ACT GAC TAC CTC ATG AAG AT-3′, beta-actin-antisense: 5′-GTC ACA CTT CAT GAT GGA GTT GAA GG-3′; humanICAM-1-sense: 5′-TTC TCG TGC CGC ACT GAA CTG-3′, humanICAM-1-antisense: 5′-GAG TCG TTG CCA TAG GTG ACT G-3′; humanIL-1β-sense: 5′-AGC TAC GAA TCT CCG ACC AC-3′, humanIL-1β-antisense: 5′-CGT TAT CCC ATG TGT CGA AGA A-3′; humanTNF-α-sense: 5′-CCT CTC TCT AAT CAG CCC TCT G-3′, humanTNF-α-antisense: 5′-GAG GAC CTG GGA GTA GAT GAG-3′; humanIL-6-sense: 5′-CCA GGA GCC CAG CTA TGA AC-3′, humanIL-6-antisense: 5′-GAT GCC GTC GAG GAT GTA CC-3′; humanIL-8-sense: 5′-AAG CTG GCC GTG GCT CTC TT-3′, humanIL-8-antisense: 5′-TGT TGG CGC AGT GTG GTC CA-3′.

### Luciferase reporter assay

Cellular lysates were subjected to a dual-luciferase reporter assay (Promega) according to the instructions of the manufacturer. Briefly, an appropriate amount of the ICAM-1 promoter luciferase reporters, together with Renilla luciferase plasmids, which served as the internal control, were cotransfected into cells. 36 h later, cellular lysates were subjected to a dual-luciferase reporter assay. The luciferase activities for the promoter reporters were detected by GloMax® 96 Microplate Luminometer (Promega) and normalized to Renilla luciferase activities. The data represented at least three independent experiments. Significant differences were analyzed using the Student’s *t* test. A value *P* < 0.05 was considered statistically significant.

### Chromatin immunoprecipitation assay

Cells were cross-linked with 1% formaldehyde at room temperature for 10 mins and then quenched using 125 mmol/L glycine. The lysate was sonicated to shear the chromatin DNA to about 500 bp.The pre-cleared chromatin solutions were incubated with specific antibodies or normal IgG for control. The immunocomplexes were captured with ssDNA/Protein A agarose beads. After extensive washing, the bound DNA fragments were eluted by heat treatment (65°C overnight) and proteinase K digestion. The eluted genomic DNA fragments were analyzed by both conventional PCR and real-time PCR. The following primer sets were used for PCR: ICAM1pro-κB-Fw: 5′- CAC TCC CAC GGT TAG CGG -3′ and ICAM1pro-κB-Rv: 5′- CCA TTT CAC AAA GCG GTA AAC -3′; CDC2pro-E2F-Fw: 5′– TGG AGG AGA GCG CTT GCG CTC GCA -3′ and CDC2pro-E2F-Rv: 5′- TTT CAA ACT CAC CGC GCT AAA GG -3′. The PCR products were separated using 2% agarose gel and visualized by ethidium bromide. The CDC2 promoter fragment acted as the positive control of bound of E2F1 [18]. For real-time PCR, relative occupancy values were calculated by determining the apparent immunoprecipitation efficiency (ratios of the amount of immunoprecipitated DNA to that of the input sample) and normalized to the level of a control sample, which was defined as 1.0. The data represented at least three independent experiments. Significant differences were analyzed using the Student’s *t* test. A value *P* < 0.05 was considered statistically significant.

### Cytokine-induced killer cells

Human peripheral blood mononuclear cells (PBMC) were separated by Ficoll density gradient centrifugation. Adherent cells were removed by adherence to plastic surfaces, Nonadherent cells were resuspended were re-suspended at 1X10^6^ cells/ml in GT-T551 medium (TAKARA, Japan) containing 10% fetal bovine serum (GIBCO, CA, USA) and cultured in the presence of immobilized anti-CD3 antibody (10 μg/10^8^ cells, BD Pharmingen, NJ, USA), recombinant human IFN-γ (1000 U/ml, R&D systems, MN, USA) and recombinant human IL-2 (1000 U/ml, R&D systems, MN, USA) for 21 days. Fresh IL-2, IFN-γ and medium were replenished every two or three days.

### Cell adhesion assay

DU145 cells were grown in 6-well tissue culture plates. 2X10^5^ Calcein-AM (Molecular Probe) labeled PBMC per well were added to the DU145 and incubated for 60 minutes in a 37°C, 5% CO_2_ incubator. After washes, cultures were fixed with 4% paraformaldehyde, and the pictures of attached monocytes were captured and counted using a fluorescence microscope at nine fields of each well.

### ^51^Cr release assay

Cytotoxicity was examined by standard chromium 51 (^51^Cr) release assays. Briefly, one million target cells (DU145 cells) were labeled with 50 μCi sodium chromate for 4 hour at 37°C and washed three times. Effector cells (CIK cells) and target cells (DU145 cells) were incubated for 4 hours at the indicated E:T ratios. The radioactivity was measured in a gamma counter. The percentage of specific lysis was calculated according to the following equation: cytotoxicity = [(sample-spontaneous)/(maximum-spontaneous)] X100%.

### Nude mouse xenograft assay

BALB/c nude mice (6–8 weeks old) were housed under specific pathogen-free condition. On day 0, 5 million DU145 cells in 200 μL of PBS were injected subcutaneously into nude mice. Tumor volumes were measured and estimated by the formula: length (mm) X width (mm) X height (mm)/2. On day 42, the mice were sacrificed and the tumor weights were measured. The animal experiments have been approved by the appropriate review board (School of Life Science, University of Science and Technology) and conform to local laws and regulations.

### Immunohistochemistry

Paraffin-embedded sections were deparaffinized, rehydrated, and subjected to antigen retrieval in 10 mmol/L citrate buffers, pH 6.0. The following primary antibodies were used: rabbit anti-E2F1 antibody (1:500) and rabbit anti-human ICAM-1 antibody (1:500). Immunoreactivity was detected using 3, 3’-diaminobenzidine substrate.

### Statistical analysis

Data are expressed as the mean value ± standard deviation (SD) of at least triplicate independent determinations for the quantitative assays in this study. Significant differences were analyzed using the Student’s *t* test. A value *P* < 0.05 was considered statistically significant. Spearman’s rank correlation coefficient was calculated by SPSS (Statistical package for the social sciences) software and used to investigate the relationship between the expression level of E2F1 and ICAM-1 using the microarray data from clinical prostate samples reported by Singh et al. [13].

## Abbreviations

ChIP: Chromatin immunoprecipitation; CIK: Cytokine-induced killer cells; E2F1: E2F transcription factor 1; GAPDH: Glyceraldehyde-3-phosphate dehydrogenase; ICAM-1: Intercellular adhesion molecule 1; IκBα: Nuclear factor kappa B inhibitor alpha; LFA-1: Lymphocyte function-associated antigen 1; NF-κB: Nuclear factor kappa B; PBMC: Peripheral blood mononuclear cells; PBS: Phosphate-buffered saline; RT-PCR: Reverse transcriptase polymerase chain reaction; shRNA: Short hairpin RNA; siRNA: Small interference RNA; WB: Western blot.

## Competing interests

The authors declare that they have no competing interests.

## Authors’ contributions

ZR and WK designed and conducted the experimental work; LW and BS, characterized the ICAM-1 mutant; JM, CZ and JS conducted and analyzed the experiments; ZT analyzed data and discussed the results; XY and WX designed the experiments, discussed the results and wrote the manuscript. All authors read and approved the final manuscript.

## References

[B1] Croci DO Salatino M Tumor immune escape mechanisms that operate during metastasis Curr Pharm Biotechnol 2011 12 1923 1936 10.2174/138920111798376987 21470132

[B2] Igney FH Krammer PH Immune escape of tumors: apoptosis resistance and tumor counterattack J Leukoc Biol 2002 71 907 920 12050175

[B3] Zamai L Rana R Mazzotti G Centurione L Di Pietro R Vitale M Lymphocyte binding to K562 cells: effect of target cell irradiation and correlation with ICAM-1 and LFA-3 expression Eur J Histochem 1994 38 Suppl 1 53 60 8547711

[B4] Slavin-Chiorini DC Catalfamo M Kudo-Saito C Hodge JW Schlom J Sabzevari H Amplification of the lytic potential of effector/memory CD8+ cells by vector-based enhancement of ICAM-1 (CD54) in target cells; implications for the intratumoral vaccine therapy Cancer Gene Ther 2004 11 665 680 10.1038/sj.cgt.7700741 15354200

[B5] Uzendoski K Kantor JA Abrams SI Schlom J Hodge JW Construction and characterization of a recombinant vaccinia virus expressing murine intercellular adhesion molecule-1: induction and potentiation of antitumor responses Hum Gene Ther 1997 8 851 860 10.1089/hum.1997.8.7-851 9143911

[B6] Koyama S Immunosuppressive effect of shedding intercellular adhesion molecule 1 antigen on cell-mediated cytotoxicity against tumor cells Jpn J Cancer Res 1994 85 131 134 10.1111/j.1349-7006.1994.tb02072.x 7908284 PMC5919416

[B7] Rosette C Roth RB Oeth P Braun A Kammerer S Ekblom J Denissenko MF Role of ICAM1 in invasion of human breast cancer cells Carcinogenesis 2005 26 943 950 15774488 10.1093/carcin/bgi070

[B8] Simmons DL The role of ICAM expression in immunity and disease Cancer Surv 1995 24 141 155 7553659

[B9] Wolfram RM Budinsky AC Brodowicz T Kubista M Köstler WJ Kichler-Lakomy C Hellan M Kahlhammer G Wiltschke C Zielinski CC Defective antigen presentation resulting from impaired expression of costimulatory molecules in breast cancer Int J Cancer 2000 88 239 244 10.1002/1097-0215(20001015)88:2<239::AID-IJC15>3.0.CO;2-Z 11004675

[B10] Johnson DG Schwarz JK Cress WD Nevins JR Expression of transcription factor E2F1 induces quiescent cells to enter S phase Nature 1993 365 349 352 10.1038/365349a0 8377827

[B11] Davis JN Wojno KJ Daignault S Hofer MD Kuefer R Rubin MA Day ML Elevated E2F1 inhibits transcription of the androgen receptor in metastatic hormone-resistant prostate cancer Cancer Res 2006 66 11897 11906 10.1158/0008-5472.CAN-06-2497 17178887

[B12] Zheng C Ren Z Wang H Zhang W Kalvakolanu DV Tian Z Xiao W E2F1 induces tumor cell survival via nuclear factor-kappaB-dependent induction of EGR1 transcription in prostate cancer cells Cancer Res 2009 69 2324 2331 10.1158/0008-5472.CAN-08-4113 19276347

[B13] Singh D Febbo PG Ross K Jackson DG Manola J Ladd C Tamayo P Renshaw AA D’Amico AV Richie JP Lander ES Loda M Kantoff PW Golub TR Sellers WR Gene expression correlates of clinical prostate cancer behavior Cancer Cell 2002 1 203 209 10.1016/S1535-6108(02)00030-2 12086878

[B14] Hou J Baichwal V Cao Z Regulatory elements and transcription factors controlling basal and cytokine-induced expression of the gene encoding intercellular adhesion molecule 1 Proc Natl Acad Sci U S A 1994 91 11641 11645 10.1073/pnas.91.24.11641 7972116 PMC45287

[B15] Ledebur HC Parks TP Transcriptional regulation of the intercellular adhesion molecule-1 gene by inflammatory cytokines in human endothelial cells. Essential roles of a variant NF-kappa B site and p65 homodimers J Biol Chem 1995 270 933 943 10.1074/jbc.270.2.933 7822333

[B16] Roebuck KA Finnegan A Regulation of intercellular adhesion molecule-1 (CD54) gene expression J Leukoc Biol 1999 66 876 888 10614768 10.1002/jlb.66.6.876

[B17] Jacobs MD Harrison SC Structure of an IkappaBalpha/NF-kappaB complex Cell 1998 95 749 758 10.1016/S0092-8674(00)81698-0 9865693

[B18] Takahashi Y Rayman JB Dynlacht BD Analysis of promoter binding by the E2F and pRB families in vivo: distinct E2F proteins mediate activation and repression Genes Dev 2000 14 804 816 10766737 PMC316494

[B19] Van Seventer GA Shimizu Y Horgan KJ Shaw S The LFA-1 ligand ICAM-1 provides an important costimulatory signal for T cell receptor-mediated activation of resting T cells J Immunol 1990 144 4579 4586 1972160

[B20] Chong AS Boussy IA Jiang XL Lamas M Graf LH Jr CD54/ICAM-1 is a costimulator of NK cell-mediated cytotoxicity Cell Immunol 1994 157 92 105 10.1006/cimm.1994.1208 7518755

[B21] Kornacker M Moldenhauer G Herbst M Weilguni E Tita-Nwa F Harter C Hensel M Ho AD Cytokine-induced killer cells against autologous CLL: Direct cytotoxic effects and induction of immune accessory molecules by interferon-gamma Int J Cancer 2006 119 1377 1382 10.1002/ijc.21994 16642465

[B22] Schmidt-Wolf IG Lefterova P Mehta BA Fernandez LP Huhn D Blume KG Weissman IL Negrin RS Phenotypic characterization and identification of effector cells involved in tumor cell recognition of cytokine-induced killer cells Exp Hematol 1993 21 1673 1679 7694868

[B23] Pievani A Borleri G Pende D Moretta L Rambaldi A Golay J Introna M Dual-functional capability of CD3 + CD56+ CIK cells, a T-cell subset that acquires NK function and retains TCR-mediated specific cytotoxicity Blood 2011 118 3301 3310 10.1182/blood-2011-02-336321 21821703

[B24] Lim CA Yao F Wong JJ George J Xu H Chiu KP Sung WK Lipovich L Vega VB Chen J Shahab A Zhao XD Hibberd M Wei CL Lim B Ng HH Ruan Y Chin KC Genome-wide mapping of RELA(p65) binding identifies E2F1 as a transcriptional activator recruited by NF-kappaB upon TLR4 activation Mol Cell 2007 27 622 635 17707233 10.1016/j.molcel.2007.06.038

[B25] Tachimori A Yamada N Sakate Y Yashiro M Maeda K Ohira M Nishino H Hirakawa K Up regulation of ICAM-1 gene expression inhibits tumour growth and liver metastasis in colorectal carcinoma Eur J Cancer 2005 41 1802 1810 10.1016/j.ejca.2005.04.036 16051479

[B26] Jochems C Schlom J Tumor-infiltrating immune cells and prognosis: the potential link between conventional cancer therapy and immunity Exp Biol Med (Maywood) 2011 236 567 579 10.1258/ebm.2011.011007 21486861 PMC3229261

[B27] Roland CL Dineen SP Toombs JE Carbon JG Smith CW Brekken RA Barnett CC Jr Tumor-derived intercellular adhesion molecule-1 mediates tumor-associated leukocyte infiltration in orthotopic pancreatic xenografts Exp Biol Med (Maywood) 2010 235 263 270 10.1258/ebm.2009.009215 20404043

[B28] Vesalainen S Lipponen P Talja M Syrjänen K Histological grade, perineural infiltration, tumour-infiltrating lymphocytes and apoptosis as determinants of long-term prognosis in prostatic adenocarcinoma Eur J Cancer 1994 30A 1797 1803 7880609 10.1016/0959-8049(94)e0159-2

[B29] Karja V Aaltomaa S Lipponen P Isotalo T Talja M Mokka R Tumour-infiltrating lymphocytes: a prognostic factor of PSA-free survival in patients with local prostate carcinoma treated by radical prostatectomy Anticancer Res 2005 25 4435 4438 16334122

[B30] Tanaka H Matsumura I Ezoe S Satoh Y Sakamaki T Albanese C Machii T Pestell RG Kanakura Y E2F1 and c-Myc potentiate apoptosis through inhibition of NF-kappaB activity that facilitates MnSOD-mediated ROS elimination Mol Cell 2002 9 1017 1029 10.1016/S1097-2765(02)00522-1 12049738

